# A new species of the rare genus *Priscomilitaris* from the Seto Inland Sea, Japan (Crustacea, Amphipoda, Priscomilitaridae)

**DOI:** 10.3897/zookeys.607.9379

**Published:** 2016-07-25

**Authors:** Ko Tomikawa, Hayato Tanaka, Takafumi Nakano

**Affiliations:** 1 Department of Science Education, Graduate School of Education, Hiroshima University, Higashi-Hiroshima 739-8524, Japan; 2 Takehara Marine Science Station, Setouchi Field Center, Graduate School of Biosphere Science, Hiroshima University, Takehara 725-0024, Japan

**Keywords:** Amphipoda, Priscomilitaridae, Priscomilitaris, new species, Seto Inland Sea, Japan, correct original spelling

## Abstract

A new species of the priscomilitarid amphipod, *Priscomilitaris heike*, from the Seto Inland Sea, Japan, is named and described. This new species is the third species of Priscomilitaridae and the second species of *Priscomilitaris*. Additionally, nucleotide sequences of nuclear 28S rRNA and histone H3 as well as mitochondrial cytochrome *c* oxidase subunit I from its holotype were determined. *Priscomilitaris heike* **sp. n.** is distinguished from its congener, *Priscomilitaris tenuis* Hirayama, 1988, by having deep antennal sinus, long flagellar article 1 of antennae 1 and 2, long mandibular palp article 2, 10 robust setae on outer ramus of maxilla 1, and rounded epimeral plates. A key to the species of Proscomilitaridae is provided.

## Introduction

Priscomilitaridae Hirayama, 1988 is a small family of amphipods comprising two monotypic genera *Priscomilitaris* Hirayama, 1988 and *Paraphotis* Ren, 1997 from coastal waters in Japan and China (Hirayama 1988; Ren 1997; Myers and Lowry 2003). *Priscomilitaris* was erected by Hirayama (1988) along with a new species *Priscomilitaris tenuis* Hirayama, 1988 from Ariake Sea, Japan. There has been no record of this genus since its original description, and thus several areas await intensive taxonomic surveys.

The Seto Inland Sea is a vast inland sea separating Honshu, Shikoku, and Kyushu. More than 90 species of amphipods were recorded from the sea (Nagata 1965; Ariyama 1996, 2015, 2016). During field surveys of marine crustaceans in the Seto Inland Sea made by HT, an undescribed species of *Priscomilitaris* was collected. In this paper, we describe and illustrate this undescribed species, and provide a key to species of Priscomilitaridae. Additionally, we provide nucleotide sequences obtained from the undescribed *Priscomilitaris* species for future molecular systematic studies.

## Material and methods

### Sample

**Figure 1.**
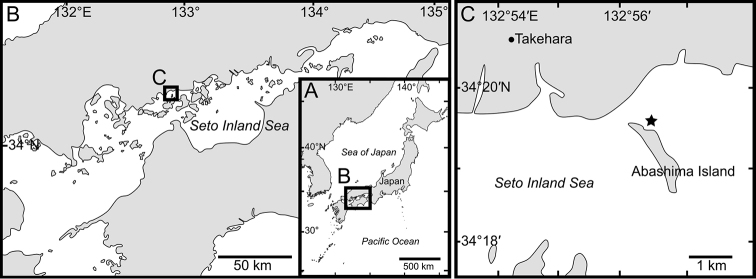
Map showing sampling locality of *Priscomilitaris heike* sp. n. **A** Japan **B** Seto Inland Sea **C** Abashima Island. Star indicates type locality.

The present specimen was collected with a dredge (mouth 40 cm wide, 15 cm high, mesh size 5 mm) at 14 m depth off Abashima Island, Takehara City, Hiroshima Prefecture, Seto Inland Sea, Japan (34°19'30.6"N, 132°56'31.9"E: Fig. 1). The specimen was preserved in 80% ethanol. For DNA extraction, dorsal side muscle was removed from inside pleon of the specimen, and was transferred into absolute ethanol.

### Morphological observation

All appendages of the examined specimen were dissected in 80% ethanol and mounted in gum-chloral medium on glass slides under a stereomicroscope (Olympus SZX7). The specimen was examined using a light microscope (Nikon Eclipse Ni) and illustrated with the aid of a camera lucida. The body length from the tip of the rostrum to the base of the telson was measured along the dorsal curvature to the nearest 0.1 mm. The nomenclature of the setal patterns on the mandibular palp follows Stock (1974). The unique holotype has been deposited in the Tsukuba Collection Center of the National Museum of Nature and Science, Tokyo (NSMT).

### PCR and DNA sequencing

The extraction of genomic DNA from pleon muscle followed Tomikawa et al. (2014). Primer sets for the PCR and cycle sequencing (CS) reactions used in this study were as follows: for 28S rRNA (28S), 28F and 28R (PCR and CS) (Hou et al. 2007) with 28SF and 28SR (CS) (Tomikawa et al. 2012) as internal primers; for histone H3 (H3), H3aF and H3bR (PCR and CS) (Colgan et al. 1998); for cytochrome *c* oxidase subunit I (COI), jgLCO1490 and jgHCO2198 (Geller et al. 2013), respectively, with M13F and M13R tails (Messing 1983), used for PCR, and then M13F and M13R used as primers for CS, followed the method outlined in Raupach et al. (2015). The PCR reactions and DNA sequencing were performed using the modified method mentioned in Nakano (2012). The PCR reactions were performed using a T100 Thermal Cycler (Bio-Rad) using an Ex *Taq* Polymerase Kit (Takara Bio Inc.) for 28S plus H3, and *Taq* Polymerase Kit (Takara Bio Inc.) for COI. The PCR mixtures were heated to 94°C for 5 min, followed by 35 cycles at 94°C (10 s each), 50°C for 28S and H3 or 49°C for COI (20 s each), and 72°C (1 min 24 s for 28S, 24 s for H3, 42 s for COI), and a final extension at 72°C for 6 min. The sequencing mixtures were heated to 96°C for 2 min, followed by 40 cycles at 96°C (10 s each), 50°C (5 s each) and 60°C (1 min for 28S, and 42 s for H3 and COI). The obtained sequences were edited using DNA BASER (Heracle Biosoft S.R.L.). These DNA sequences were deposited with the International Nucleotide Sequence Database Collaboration (INSDC) through the DNA Data Bank of Japan (DDBJ).

## Taxonomy

### Family Priscomilitaridae

Taxon classificationAnimaliaAmphipodaPriscomilitaridae

Hirayama, 1988

#### Remarks.

This family name was subsequently used as Priscomilitariidae by Myers and Lowry (2003). The generic name of its type species, *Priscomilitaris*, ends in a Latin word, militaris (genitive militaris, stem militar-). Therefore, the stem of this family name should be Priscomilitar- according to the Art 29.3. of the Code (ICZN 1999). The original spelling by Hirayama (1988) is thus obviously correct. Because Myers and Lowry (2003) did not provide a statement for any demonstrably intentional change of the spelling Priscomilitaridae, the spelling Priscomilitariidae is an incorrect subsequent spelling according to the Art 33.3. of the Code. This incorrect spelling is used in the influential web sources, e.g. WoRMS (Horton and Lowry 2015). The incorrect spelling of Priscomilitaridae on those web registries should be emended to avoid additional erroneous citations of the spelling of this family name.

### Genus *Priscomilitaris* Hirayama, 1988

#### Priscomilitaris heike sp. n.

Taxon classificationAnimaliaAmphipodaPriscomilitaridae

http://zoobank.org/4F6D58AC-1993-40C0-B0C2-B2CBADE15140

New Japanese name: Heike-yokoebi

**Figure 2.**
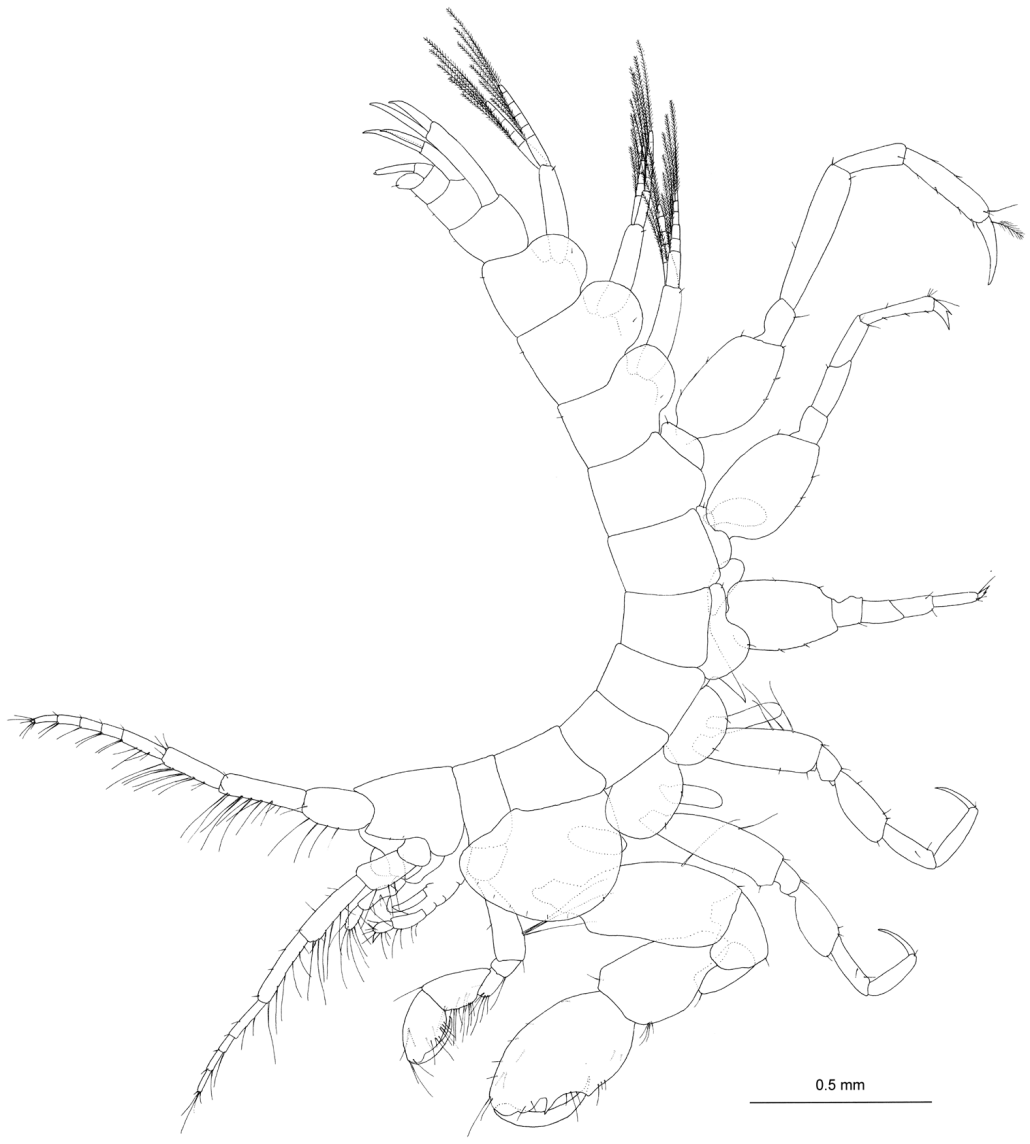
*Priscomilitaris heike* sp. n., holotype, male, 2.3 mm, NSMT-Cr 24368, Abashima Island, Takehara, Hiroshima Prefecture, Japan. Habitus, lateral view.

**Figure 3.**
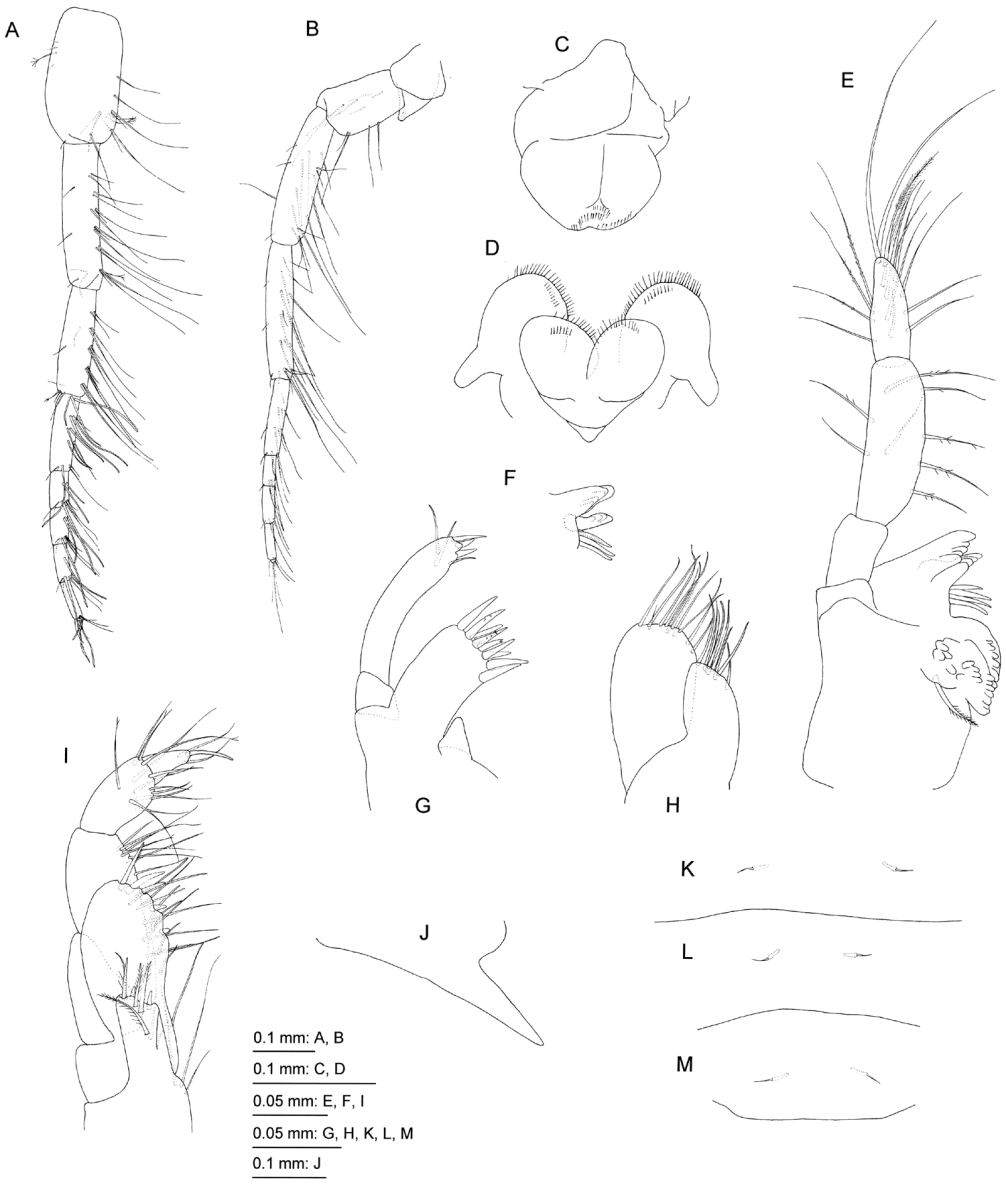
*Priscomilitaris heike* sp. n., holotype, male, 2.3 mm, NSMT-Cr 24368, Abashima Island, Takehara, Hiroshima Prefecture, Japan. **A** antenna 1, medial view **B** antenna 2, medial view **C** upper lip, anterior view **D** lower lip, ventral view **E** left mandible, medial view **F** incisor, lacinia mobilis, and accessory setal row of right mandible, lateral view **G** maxilla 1, anterior view **H** maxilla 2, anterior view **I** maxilliped, anterior view **J** sternal tooth on pereonite 5, right lateral view **K–M** urosomites 1–3, dorsal views.

**Figure 4.**
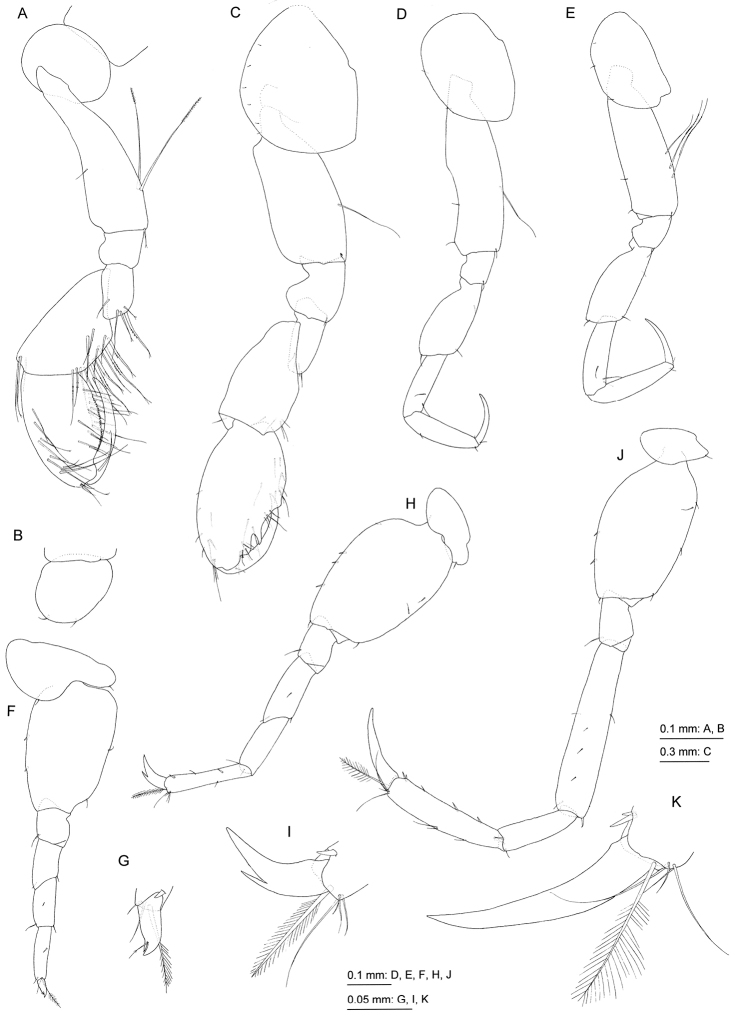
*Priscomilitaris heike* sp. n., holotype, male, 2.3 mm, NSMT-Cr 24368, Abashima Island, Takehara, Hiroshima Prefecture, Japan. **A** right gnathopod 1, medial view **B** coxa of left gnathopod 1, lateral view **C** gnathopod 2, lateral view **D** pereopod 3, lateral view **E** pereopod 4, lateral view **F** pereopod 5, lateral view **G** distal part of propodus and dactylus of pereopod 5, lateral view **H** pereopod 6, lateral view **I** distal part of propodus and dactylus of pereopod 6, lateral view **J** pereopod 7, lateral view **K** distal part of propodus and dactylus of pereopod 7, lateral view.

**Figure 5.**
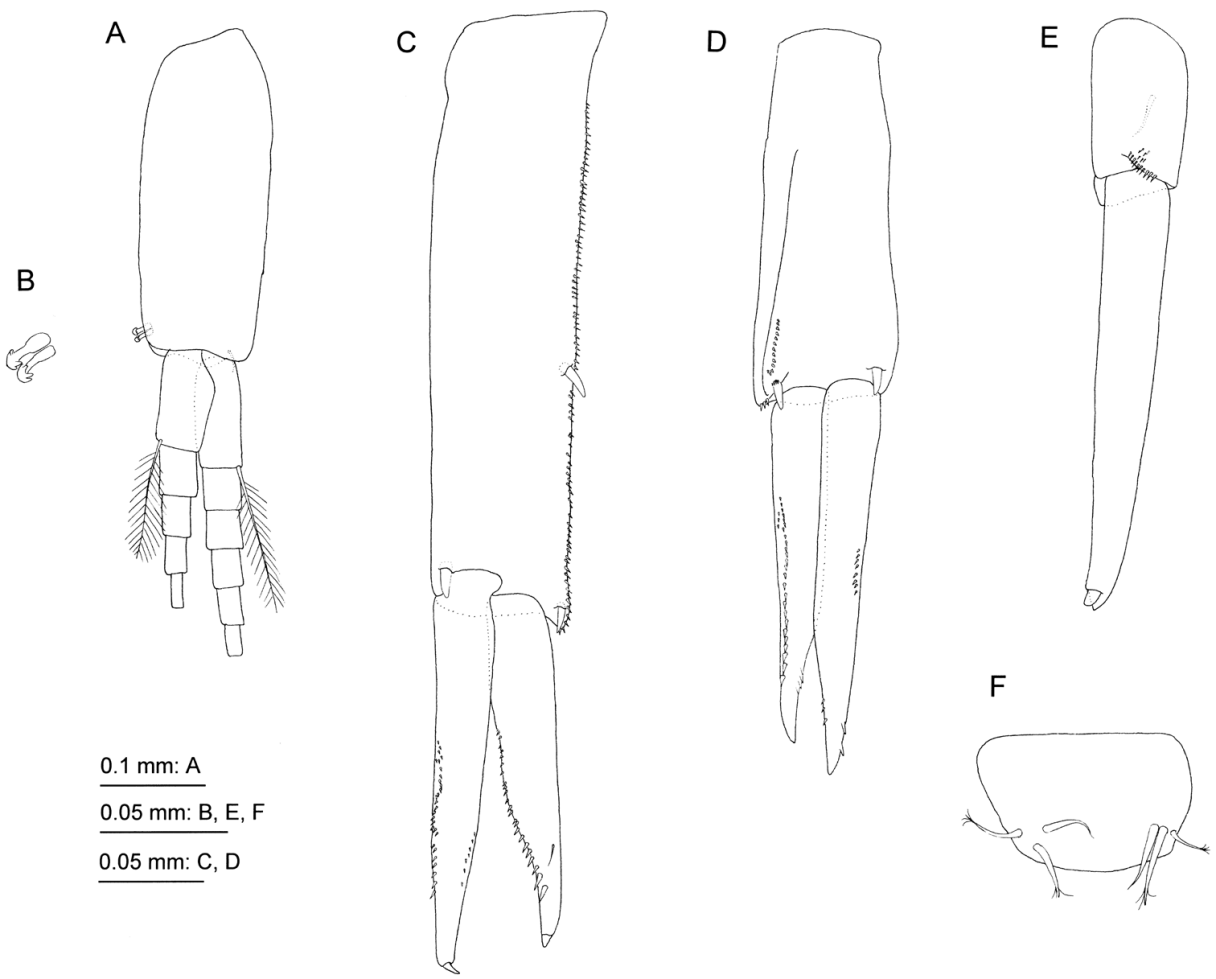
*Priscomilitaris heike* sp. n., holotype, male, 2.3 mm, NSMT-Cr 24368, Abashima Island, Takehara, Hiroshima Prefecture, Japan. **A** pleopod 2, posterior view, some setae on rami omitted **B** retinacula on peduncle of pleopod 2, posterior view **C–E** uropods 1–3, dorsal views **F** telson, dorsal view.

Figures 2 , 3 , 4 , 5

##### Holotype.

Male (2.3 mm), NSMT-Cr 24368, east of Abashima Island (34°19'30.6"N, 132°56'31.9"E; 14 m deep), Takehara, Hiroshima, Japan, 15 February 2016, collected by H. Tanaka.

##### Description.

Head (Fig. 2): slightly shorter than pereonites 1 and 2 combined; rostrum short, acute; eyes absent; lateral cephalic lobe acute, ventral margin with 2 minute setae; antennal sinus rounded. Pereon (Fig. 2): pereonite 1 short, 0.6 times as long as pereonite 2; pereonite 5 with strong sternal tooth extending anteroventrally (Fig. 3J). Pleon (Fig. 2): dorsal surfaces of pleonites 1–3 smooth, with pair of minute setae; epimeral plates 1–3 rounded, each with minute seta on ventral submargin. Urosomites 1–3 (Figs 2, 3K–M): dorsal surfaces with pair of minute setae.

Antenna 1 (Fig. 3A): length 0.4 times as long as body; length ratio of peduncle articles 1–3 1.0 : 1.1 : 0.8; peduncle article 1 with 1 penicillate and 3 minute setae on anterior margin, and 2 pairs of setae and 2 single setae on posterior margin; peduncle article 2 with 2 setae on medial surface, and 3 pairs and 3 clusters of setae on posterior margin; peduncle article 3 medial and lateral surfaces each with a seta, and 3 pairs and 2 clusters of setae on posterior margin; primary flagellum 6-articulate with long aesthetascs, article 1 long, length 2.1 times as long as article 2, article 6 minute; accessory flagellum absent.

Antenna 2 (Fig. 3B): length 0.9 times as long as antenna 1; length ratio of peduncular articles 3–5 1.0 : 1.7 : 1.7; article 3 quadrate with 2 single setae and a pair of setae on posterior margin; article 4, anterior margin with 2 short setae and one long seta, posterior margin with 4 single setae and pair of setae; article 5 with 2 short setae on anterior margin, and 4 pairs and 2 clusters of setae on posterior margin; flagellum 5-articulate, article 1 long, length 2.5 times as long as article 2, article 5 minute; calceoli absent.

Upper lip (Fig. 3C): ventral margin concave, with minute setae. Lower lip (Fig. 3D): outer lobes broad, setulose, mandibular lobes narrow; inner lobes distinct. Mandible (Fig. 3E, F): left and right mandibles similar to each other; incisors 5-dentate, lacinia mobilis 4-dentate, accessory setal rows each with 4 blade setae, molar process triturative with a plumose seta; palp 3-articulate, length ratio of article 1–3 1.0 : 2.1 : 1.3, article 1 bare, article 2 with 4 ventral, 2 dorsal and 2 lateral setae, article 3 with 4 A-, 4 C-, 9 D-, and 2 Esetae. Maxilla 1 (Fig. 3G): inner plate small, subtriangular without setae; outer plate rectangular with 10 weakly serrate or unarmed robust setae; palp 2-articulate, exceeding outer plate, article 1 lacking setae, article 2 with 3 robust and 1 slender setae on apical margin, and 2 slender setae on apical submargin. Maxilla 2 (Fig. 3H): inner and outer plates with apical setae. Maxilliped (Fig. 3I): inner plate rectangular, not reaching half of palp article 1, with 2 robust setae on apical margin; outer plate weakly curved inward, exceeding half of palp article 2, with robust and slender setae; palp 4-articulate, ventral margin of article 2 with setae, medial and lateral surfaces of article 3 with setae, article 4 with long, slender robust setae.

Gnathopod 1 (Fig. 4A, B): smaller than gnathopod 2, coxa ovate, with or without ventral setae; posterior margin of basis with long setae; carpus not lobate, slightly longer than propodus, with weakly pectinate setae on posterior margin; propodus ovate, posterior margin serrate; dactylus long, smooth. Gnathopod 2 (Fig. 4C): coxa semicircular, covering coxa of gnathopod 1, with minute setae on ventral submargin; basis anteroproximally concave, posterior margin with a long seta; carpus not lobate, length 0.9 times as long as propodus; palmar margin of propodus shallowly concave, with acute protuberance; dactylus long, smooth, exceeding palmar margin.

Pereopod 3 (Fig. 4D): coxa semioval with 2 minute setae on ventral margin; anterodistal margin of basis shallowly concave, posterior margin with long seta; length ratio of merus, carpus, propodus and dactylus 1.0 : 0.8 : 0.9 : 0.6; dactylus smooth. Pereopod 4 (Fig. 4E): coxa semioval with 3 minute setae on ventral margin; basis lacking anterodistal concavity, posterior margin with 3 long setae; length ratio of merus, carpus, propodus and dactylus 1.0 : 0.8 : 0.9 : 0.6; dactylus smooth. Pereopod 5 (Fig. 4F, G): coxa bilobate, anterior lobe large with small seta on distal margin, posterior lobe with small seta on posterodistal corner; basis subrectangular, lacking posterodistal lobe; length ratio of merus, carpus, propodus and dactylus 1.0 : 1.0 : 1.0 : 0.4; propodus with long plumose seta on distal margin; dactylus with small accessory tooth. Pereopod 6 (Fig. 4H, I): coxa shallow, bilobate, posterior lobe with small seta on posterodistal corner; basis oval, lacking posterodistal lobe; length ratio of merus, carpus, propodus and dactylus 1.0 : 0.8 : 1.0 : 0.3; distal margin of merus oblique; propodus with long plumose seta on distal margin; dactylus with accessory tooth. Pereopod 7 (Fig. 4J, K): coxa oblong with seta on posterodistal corner; basis ovate, lacking posterodistal lobe; ischium rectangular, length 1.6 times as long as width; length ratio of merus, carpus, propodus and dactylus 1.0 : 0.5 : 0.7 : 0.5; propodus with long plumose seta on distal margin; dactylus smooth.

Coxal gills (Fig. 2): present on gnathopod 2, pereopods 3–6.

Pleopods 1–3 (Fig. 5A, B) each with paired retinacula (Fig. 5B) on inner distal margin of peduncle, bifid plumose setae (clothes-pin setae) on inner basal margin of inner ramus absent; inner and outer rami of pleopods 1–3 consisting of 5 and 6 articles, respectively.

Uropod 1 (Fig. 5C): extending beyond uropod 2; peduncle long, length 1.6 times as long as inner ramus, dorsolateral margin with robust seta and numerus minute setae; inner ramus length 1.1 times as long as outer ramus, inner and outer margins with minute robust setae, apical part with robust seta; inner margin of outer ramus with minute robust setae, outer submargin with seta, apical part with robust seta. Uropod 2 (Fig. 5D): extending beyond uropod 3; peduncle almost as long as inner ramus, distal part of dorsolateral margin with minute robust setae; inner ramus slightly longer than outer ramus, inner distal and outer margins with minute robust setae; outer ramus with minute robust setae on outer and inner distal margins. Uropod 3 (Fig. 5E): extending beyond telson, uniramous; peduncle short, with facial seta and minute robust setae along with distal margin; ramus long, length 2.4 times as long as peduncle, 1-articulate with terminal robust seta. Telson (Fig. 5F): entire, fleshy, length 0.6 times width, with 2 clusters of 6 setae on distal submargin. Female unknown.

##### Sequences.

Three nucleotide sequences of the holotype, NSMT-Cr 24368, were determined: 28S, LC155260 (1274 bp); H3, LC155261 (328 bp); and COI, LC155259 (658 bp).

##### Distribution.

This species is known only from the type locality.

##### Etymology.

After ‘Heike’ (literally ‘House of Taira’) that controlled the Seto Inland Sea, the Chugoku region, the Shikoku region as well as the Kyushu region during the Heian Period. The specific name is a Japanese word, not a Latin or Latinized one.

##### Remarks.

*Priscomilitaris heike* sp. n. is distinguished from *Priscomilitaris tenuis* by the following features (features of *Priscomilitaris tenuis* in parentheses): antennal sinus deep (shallow), flagellar article 1 of antenna 1 length 2.1 (1.3) times as long as article 2, flagellar article 1 of antenna 2 length 2.5 (1.0) times as long as article 2, mandibular palp article 2 longer than article 3 (subequal), outer plate of maxilla 1 with 10 (12) robust setae, epimeral plates rounded (quadrate). This new species differs from *Paraphotis sinensis* in the following features (features of *Paraphotis sinensis* in parentheses): antennal sinus deep (shallow), sternal tooth present on pereonite 5 (pereonite 4), flagellar article 1 of antenna 1 length 2.1 (1.4) times as long as article 2, flagellar article 1 of antenna 2 length 2.5 (1.4) times as long as article 2, outer plate of maxilla 1 with 10 (9) robust setae, palmar margin of propodus of gnathopod 2 with protuberance (absent).

#### Key to species of Priscomilitaridae

**Table:** 

1	Gnathopod 2, palmar margin of propodus without protuberance	***Paraphotis sinensis* Ren, 1997**
‒	Gnathopod 2, palmar margin of propodus with protuberance	**2**
2	Antennal sinus shallow, flagellar article 1 of antennae 1 and 2 length subequal to or weakly longer than article 2, mandibular palp article 2 subequal to article 3, outer plate of maxilla 1 with 12 robust setae, epimeral plates quadrate	***Priscomilitaris tenuis* Hirayama, 1988**
‒	Antennal sinus deep, flagellar article 1 of antennae 1 and 2 length more than twice as long as article 2, mandibular palp article 2 longer than article 3, outer plate of maxilla 1 with 10 robust setae, epimeral plates rounded	***Priscomilitaris heike* sp. n.**

## Supplementary Material

XML Treatment for Priscomilitaridae

XML Treatment for Priscomilitaris heike
